# Sequential damage assessment of the posterolateral complex of the knee joint: a finite element study

**DOI:** 10.1186/s13018-022-03034-3

**Published:** 2022-03-28

**Authors:** Cong-Cong Wu, Li-Mei Ye, Xiao-Fei Li, Lin-Jun Shi

**Affiliations:** 1 Department of Orthopeadics, Affiliated Jinhua Hospital, Zhejiang University School of Medicine, No. 365 Renmin East Road, Jinhua City, 321000 Zhejiang Province China; 2 Department of Radiology, Affiliated Jinhua Hospital, Zhejiang University School of Medicine, No. 365 Renmin East Road, Jinhua City, 321000 Zhejiang Province China

**Keywords:** Finite element, Posterolateral complex, Tibial displacement, Tibial external rotation, Tibial varus angulation, Instantaneous axis of rotation

## Abstract

**Background:**

The posterolateral complex (PLC), which consists of the popliteus tendon (PT), lateral collateral ligament (LCL), and popliteofibular ligament (PFL), is an indispensable structure of the knee joint. The aim of this study was to explore the functionality of the PLC by determining the specific role of each component in maintaining posterolateral knee stability.

**Methods:**

A finite element (FE) model was generated based on previous material property data and magnetic resonance imaging of a volunteer’s knee joint. The injury order of the PLC was set as LCL, PFL, and PT. A combined compressive load of 1150 N and an anterior tibial load of 134 N was applied to the tibia to investigate tibial displacement (TD). Tibial external rotation (TER) and tibial varus angulation (TVA) were measured under bending motions of 5 and 10 Nm. The instantaneous axis of rotation (IAR) of the knee joint under different rotation motions was also recorded.

**Results:**

The TD of the intact knee under a combined compressive load of 1150 N and an anterior tibial load of 134 N matched the values determined in previous studies. Our model showed consistent increases in TD, TVA, and TER after sequential damage of the PLC. In addition, sequential disruption caused the IAR to shift superiorly and laterally during varus rotation and medially and anteriorly during external rotation. In the dynamic damage of the PLC, LCL injury had the largest effect on TD, TVA, TER, and IAR.

**Conclusions:**

Sequential injury of the PLC caused considerable loss of stability of the knee joint according to an FE model. The most significant structure of the PLC was the LCL.

## Introduction

The posterolateral complex (PLC), also referred to as the posterolateral corner or posterolateral structure, plays an indispensable role in maintaining the stability of the knee joint. Previous research showed that the PLC comprises several ligaments and tendons, with the main structures being the popliteus tendon (PT), popliteofibular ligament (PFL), and lateral collateral ligament (LCL) [1–3]. Although PLC damage may be isolated, such as injury to a cruciate ligament or tibial plateau fracture, PLC failure is an area of increasing research interest. However, identifying PLC damage is often clinically challenging and mostly based on indirect measures, including clinical tests such as the passive external rotational and posterolateral drawer tests [4]. Impingement fractures of the anteromedial tibial margin can be seen on radiography, and their detection can facilitate the diagnosis of PLC injury [5]. Magnetic resonance imaging has been used to accurately identify PLC injury and reveal many of its features, thus allowing better surgical planning [6–8]. Failure to diagnose PLC injury promptly or directly may lead to chronic joint pain, cartilage degeneration, or failure of cruciate ligament reconstruction. PLC reconstruction to restore the stability of the posterolateral knee joint after PLC failure has been proposed based on clinical experience. Previous studies have shown that patients undergoing reconstruction of PLC structures achieve good functional outcomes.

The stability of the knee is relatively complex, for the stability is achieved by several structures. The anterolateral ligament and anterior cruciate ligament help to maintain the anterolateral knee stability [9–11]. But in the posterolateral part of the knee, the PLC plays a significant role in maintaining the stability. The structure of the PLC has been examined in cadaver studies, which identified main structures in this area: the LCL, PFL, and PT [12, 13]. Other cadaver studies investigated knee joint stability by measuring tibial displacement (TD) in the anterior direction, tibial external rotation (TER), and tibial varus angulation (TVA) [14–18]. Finite element (FE) models have been extensively used to evaluate trauma leading to structural failure. The advantages of FE models over cadaver studies include their repeatability and convenience. However, while instability of the knee joint after PLC failure has been determined in cadaver studies, sequential damage of the PLC has been investigated in only a few FE models. In addition, the instantaneous axis of rotation (IAR), examined by sequentially cutting the LCL, PFL, and PT, has not been used to evaluate posterolateral knee stability. Thus, the aim of this study was to establish a FE model in which the PLC was sequentially damaged, to evaluate its biomechanical role in maintaining the stability of the knee joint, as assessed based on TD, TER, TVA, and the IAR.

## Materials and methods

The study was approved by our Institutional Research Ethics Committee (Affiliated Jinhua Hospital, Zhejiang University School of Medicine, People’s Republic of China; approval no. 2021-099-001). Knee measurements were made in a male volunteer (age: 28 years, height: 175 cm, weight: 67 kg) with no previous history of osteoarthritis or fracture. He provided informed consent after being informed about the research. Magnetic resonance imaging scans of his full-extension knee were obtained in our radiology department and used to construct a three-dimensional knee joint FE model. Mimics software (version 20.0) was used to manage the magnetic resonance scan data and obtain a simplified model of the knee joint. The data were then imported into Geomagic software (2012) to construct a solid skeletal model, and SolidWorks software (2015) was used to add meniscus and articular cartilage to the model. Ligaments and tendons were then added using ANSYS software (version 18.0). The latter software was also used to assess the final model of sequential PLC damage.

### Material properties

**Fig. 1:**
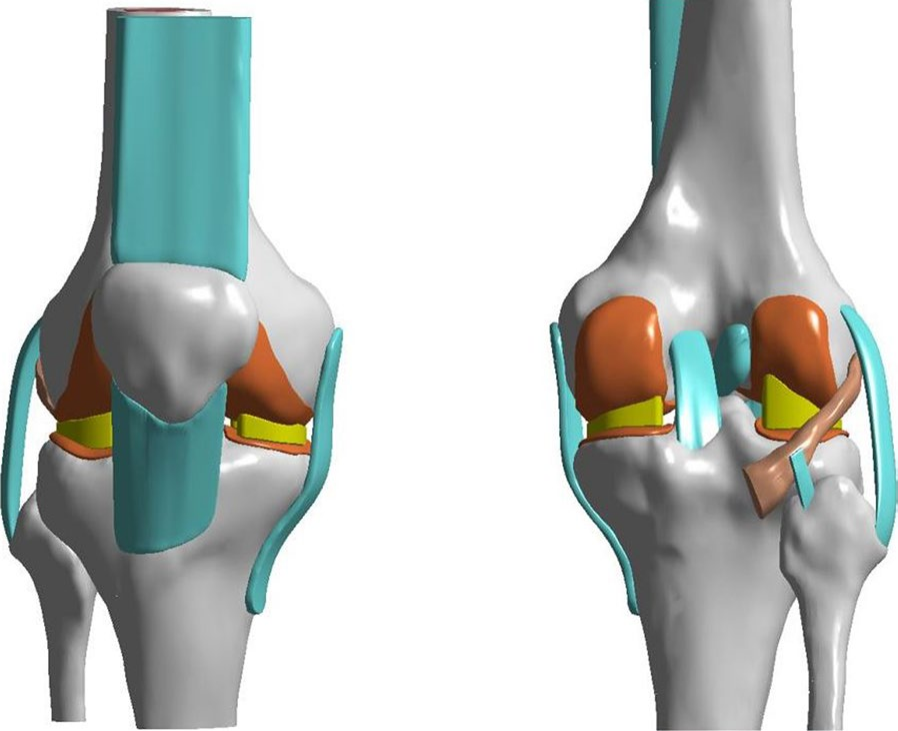
Finite element model of the knee joint

As Fig. 1 shows, the knee is composed of bony structures (femur, tibia, fibula, and patella), articular cartilage, menisci, ligaments (anterior cruciate ligament, posterior cruciate ligament, medial collateral ligament, LCL, and PFL), and tendons (quadriceps tendon, patellar tendon, and PT). Table 1 lists the materials used in our modeled knee [19–22]. Bones, articular cartilage, menisci, ligaments, and tendons were modeled to behave as elastic materials. Cortical and cancellous bone was modeled as tetrahedral block-structured meshes. Menisci, articular cartilage, ligaments, and tendons were constructed from hexahedral block-structured meshes. The bony structures, articular cartilage, menisci, ligaments, and tendons were then integrated as 174,265 nodes and 571,228 solid elements.

**Table 1:** The material properties of the finite element model

Component name	Young’s modulus (MPa)	Poisson’s ratio
Cortical bone	17,000	0.3
Cancellous bone	400	0.3
Articular cartilage	13	0.4
Meniscus	35	0.3
Patellar tendon	336	0.4
Quadriceps tendon	370	0.4
Anterior cruciate ligament	123	0.4
Posterior cruciate ligament	168	0.4
Medial collateral ligament	224	0.4
Lateral collateral ligament	280	0.4
Popliteofibular ligament	131	0.4
Popliteus tendon	25	0.4

MPa, megapascal

### Boundary and loading conditions

In the biomechanical evaluation, the freedom of movement of the proximal surfaces of the femur, quadriceps tendon, and of the distal surface of the PT, was completely restricted. The external periphery and two horns of the menisci were defined as attached to the tibial plateau. As in a previous study, the friction coefficient between the menisci and articular cartilages was 0.02 [23]. For the interaction of the articular cartilages between the patellofemoral and tibiofemoral joints, the friction coefficient was set to 0.02 [23]. By contrast, the upper tibiofibular joint was defined as bound. The proximal and distal ends of ligaments and tendons, except for the proximal end of the quadriceps tendon and distal end of the PT, were bonded to bone. For validation analysis, a combined compressive load of 1150 N and an anterior load of 134 N was applied to the tibia to allow comparison between the kinematics determined in this study and those reported in previous research. In the following biomechanical test, pure bending motions of 5 and 10 Nm were applied at the tibia to evaluate external rotation and varus angulation.

### Data analysis

To mimic sequential reduction of the PLC, structural failure was simulated in the following order: LCL, PFL, and PT. According to published studies, TD was defined according to the distance of anterior motion by the tibia, while IAR was defined as the intersection between the perpendicular bisectors of the translation vectors of two peripheral points of the tibial plateau. TER and TVA were defined according to the degree of rotation of the IAR relative to the angle achieved under bending motion. Statistical analysis in this study was performed by using SPSS (version 20.0) and Stata (version 12.0), and the *p* value was set < 0.05. One-sample T test was applied to compare the difference between the TD in intact knee and that in previous studies. One-way Analysis of Variance was applied for statistical comparison of TD, TVA, TER, IAR in different stages of PLC damage.

## Results

### Analysis of TD

#### Analysis of the intact model

Table 2 shows the kinematics of the intact knee under a combined compressive load of 1150 N and a 134 N anterior load, as determined in the current and previous studies [14–16]. Peña et al. [14] built a FE model to evaluate the ligaments in the healthy human knee joint and determined that the anterior translation of intact tibia was 4.75 mm under a combined compressive load of 1150 N and a 134 N anterior load. In the cadaver experiments conducted by Gabriel et al. [15], the anterior translation of tibia under under a combined compressive load of 1150 N and a 134 N anterior load was 4.0 ± 1.0 mm at full extension. In the current study, the mean TD was 4.62 ± 0.3 mm.

**Table 2:** Comparison of the kinematics of intact knee with published experimental results under a combined compressive load of 1150 N and an anterior tibial load of 134 N

	Anterior translation (mm)	Medial translation (mm)	Distal translation (mm)	Valgus rotation (°)	Internal rotation (°)
Current study	4.62 ± 0.2	0.64 ± 0.1	0.11 ± 0.2	− 0.26 ± 0.1	1.48 ± 0.1
Peña	4.75	0.56	− 1.10	0.76	1.60
Gabriel	4.0 ± 1.0	0.6 ± 0.6	0.3 ± 0.3	− 0.2 ± 0.7	1.7 ± 1.5

mm, millimeter, °, degree

#### Analysis of models with PLC failure

**Fig. 2:**
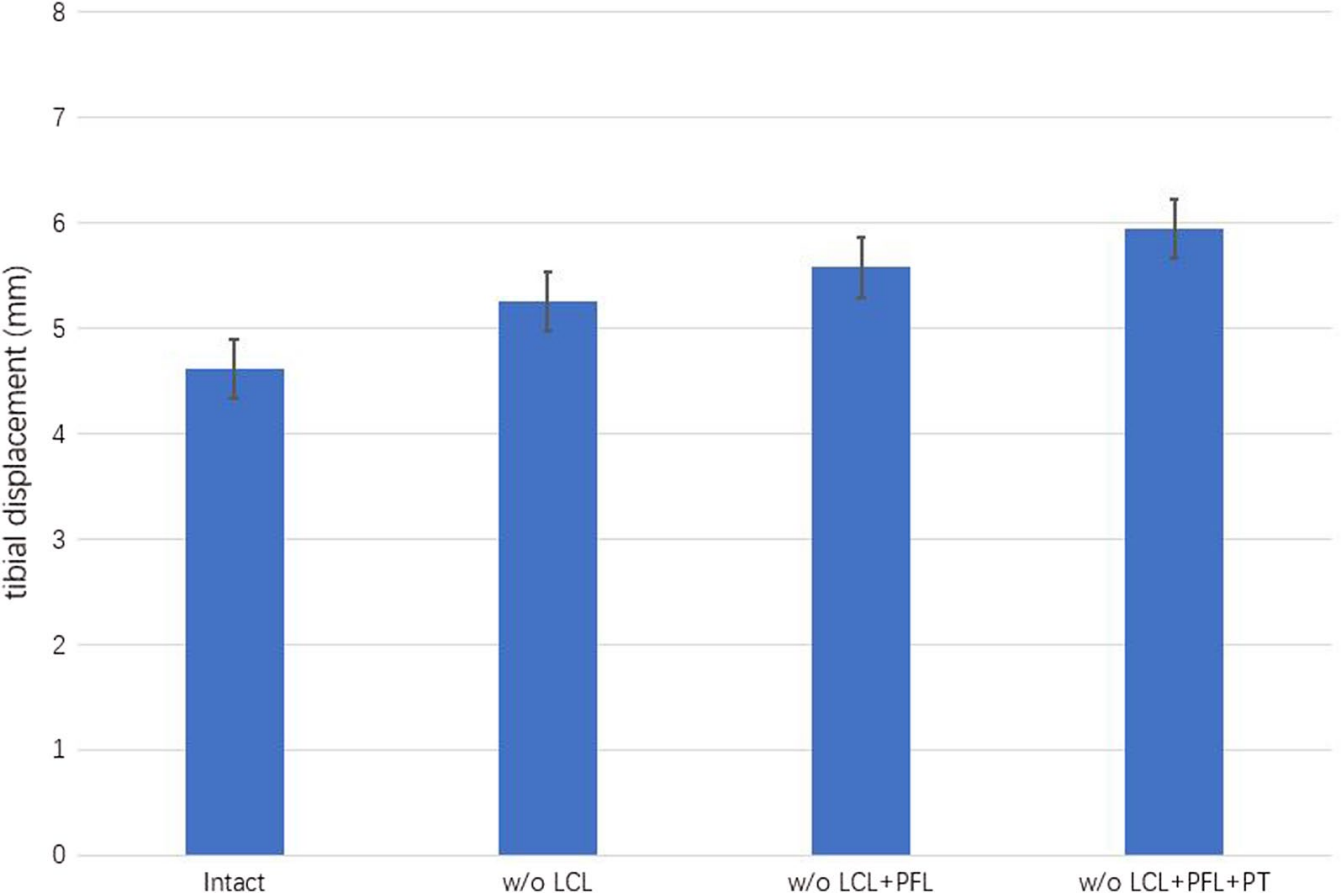
The mean TD under a combined compressive load of 1150 N and an anterior tibial load of 134 N during sequential cutting of the posterolateral complex (PLC), as follows: LCL, PFL, and PFL. TD, tibial displacement; w/o, without; LCL, lateral collateral ligament; PFL, popliteofibular ligament; PT, popliteus tendon

The changes in TD along the anterior direction under a combined compressive load of 1150 N and a 134 N anterior load and various structural disruptions of the PLC are shown in Fig. 2. TD ranged from 5.38 to 6.11 mm when the PLC was sequentially cut. PLC disruption resulted in slight increases in TD, with LCL failure having the largest influence (increase of TD = 0.76 mm), followed by the PT (0.44 mm) and PFL (0.29 mm).

### Analysis of TVA

**Fig. 3:**
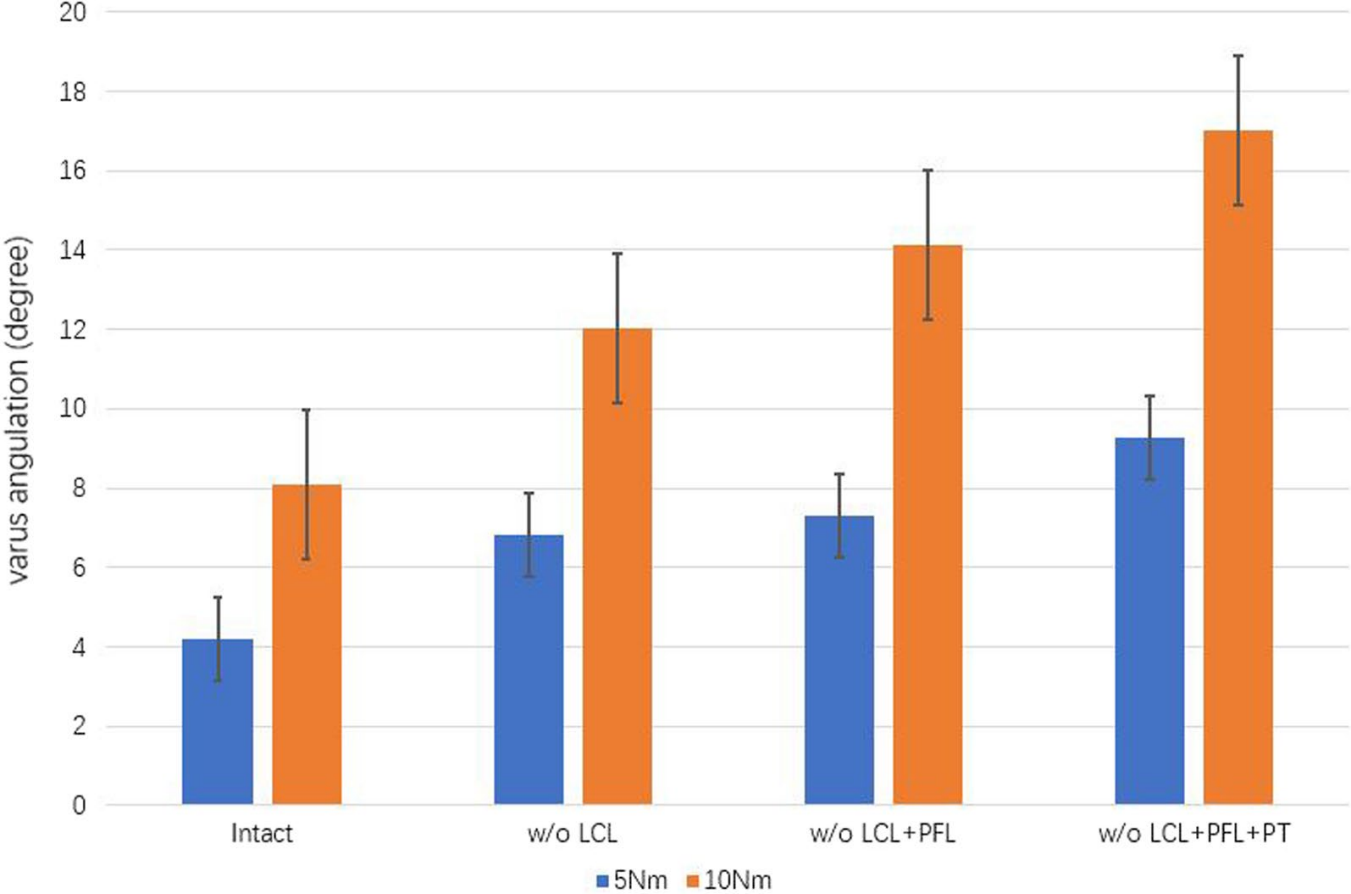
The mean TVA under 5 and 10 Nm of pure motion during sequential cutting, as follows: LCL, PFL, and PT. TVA, tibial varus angulation; w/o, without; LCL, lateral collateral ligament; PFL, popliteofibular ligament; PT, popliteus tendon

**Fig. 4:**
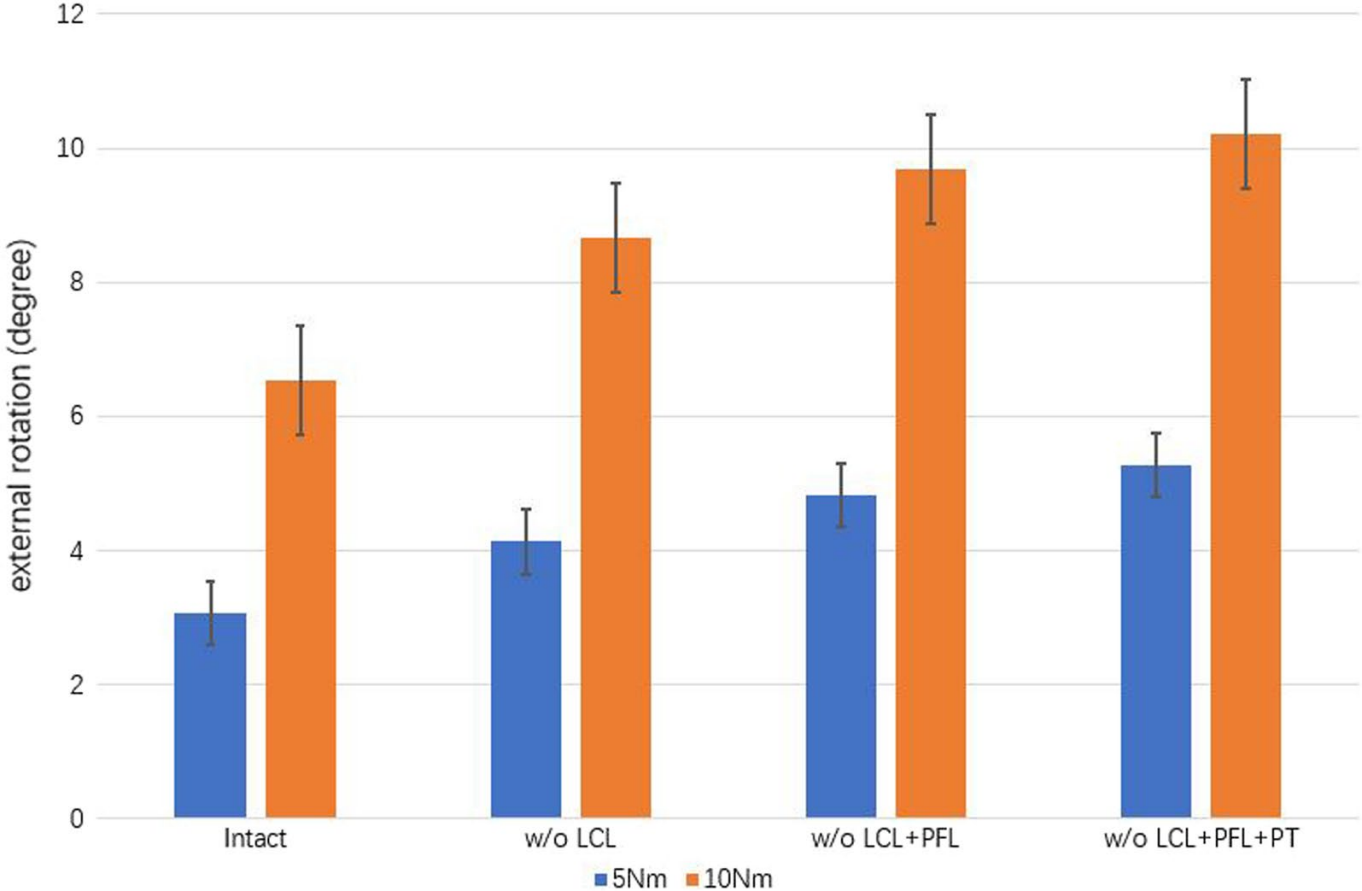
The mean TER under 5 and 10 Nm of pure motion during sequential cutting, as follows: LCL, PFL, and PT. TER, tibial external rotation; w/o, without; LCL, lateral collateral ligament; PFL, popliteofibular ligament; PT, popliteus tendon

The changes in TVA under pure bending motions of 5 and 10 Nm during sequential disruption of the PLC are shown in Fig. 3. Under 5 Nm varus motion, the mean TVA was 4.21 ± 0.2° in the intact model, 6.83 ± 0.5° when the LCL was cut, 7.29 ± 0.4° when the PFL was cut, and 9.29 ± 0.3° when the PT was cut. Under 10 Nm varus motion, the mean TVA was 8.09 ± 0.8°, 12.04 ± 0.6°, 14.13 ± 0.5°, and 17.03 ± 0.8°, respectively. The difference between groups was significant (*p* < 0.05). Figure 4 also shows that the LCL had the most significant influence on TVA, under bending motions of both 5 and 10 Nm. Failures of the PFL and PT had less impact on TVA.

### Analysis of TER

The changes in TER under pure bending motions of 5 and 10 Nm during sequential disruption of the PLC are shown in Fig. 4. The mean TER in the intact model was 3.07 ± 0.4° at a bending motion of 5 Nm and 6.54 ± 0.9° at a bending motion of 10 Nm. Slight increases in the TER were observed under a 5 Nm bending motion and LCL, PFL, and PT failure, of 1.07°, 0.69°, and 0.45°, respectively. Under 10 Nm bending motion, the corresponding increases of TER values were 2.12°, 1.03°, and 0.52°. The differences between the groups were significant (*p* < 0.05). LCL damage had a larger effect on TER than either PFL or PT damage, under both 5 and 10 Nm bending motions.

### Analysis of the IAR

#### IAR in the varus rotation model

**Fig. 5:**
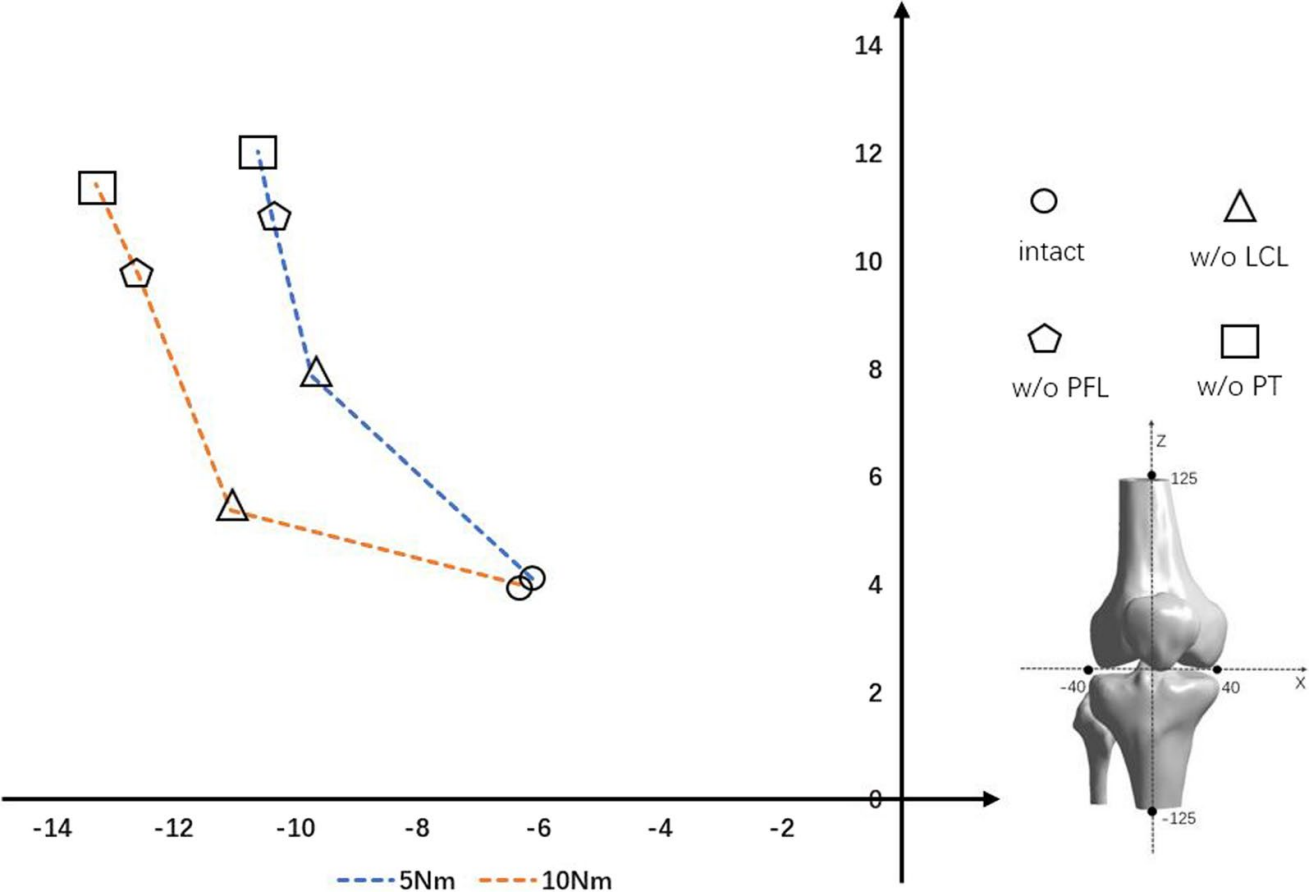
Trajectory of the IAR under 5 and 10 Nm bending motion (varus rotation) during sequential cutting, as follows: LCL, PFL, and PT. IAR, instantaneous axis of rotation; +, values for the interior and superior directions; −, values for the exterior and inferior directions. Values for the coordinate system are in millimeters

As shown in Fig. 5, the IAR gradually approached the lateral femoral condyle under both 5 and 10 Nm varus rotation. Under 5 Nm varus rotation, sequential reduction of the structures of the PLC caused the IAR to shift superiorly and laterally, whereas under 10 Nm bending motion the shift was slightly lateral. Cutting the LCL caused a shift in the IAR of 5.21 mm under 5 Nm bending motion and 4.93 mm under 10 Nm bending motion. Cutting the PFL and PT caused only a slight shift in the position of the IAR.

#### IAR in the external rotation model

**Fig. 6:**
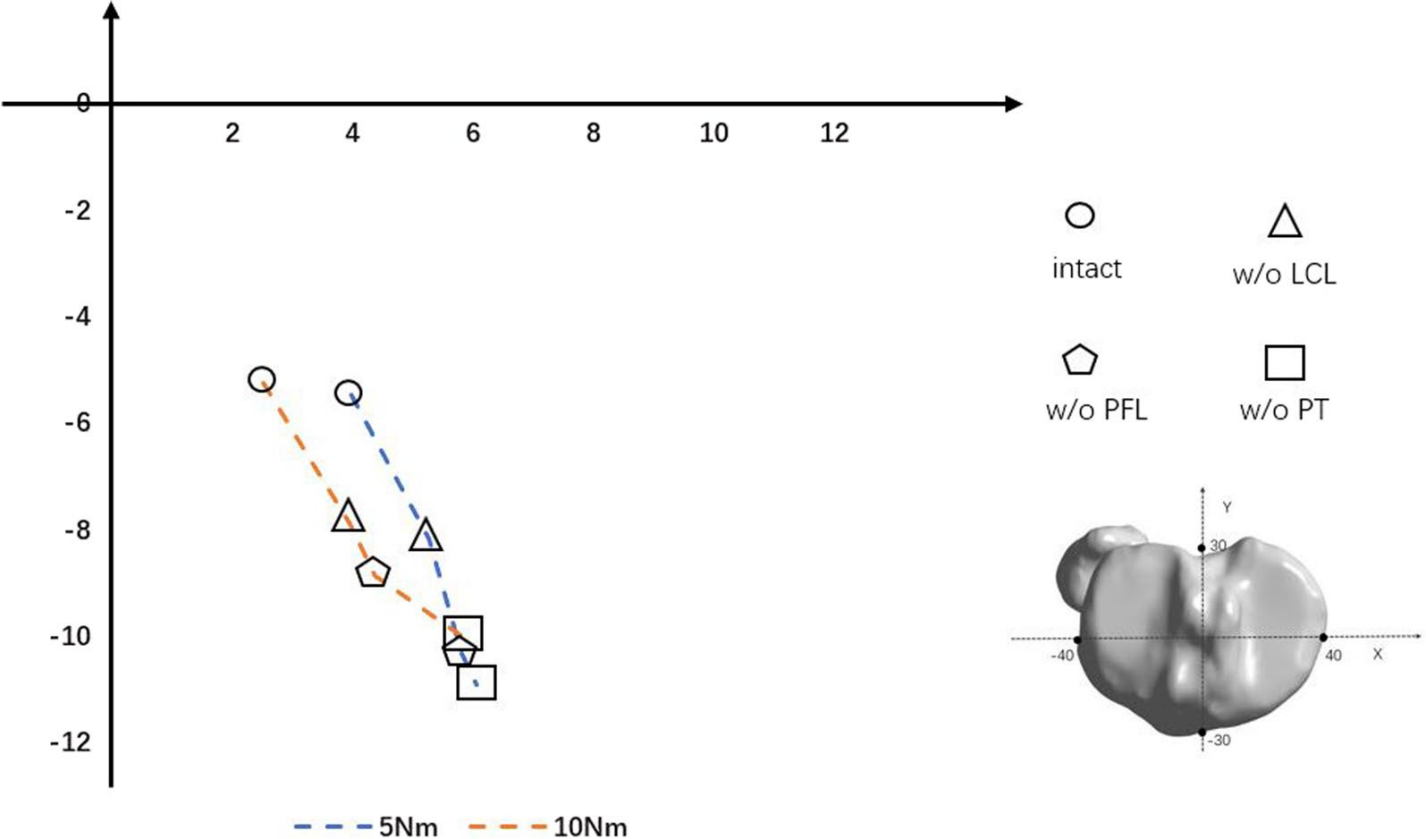
Trajectory of the IAR under 5 and 10 Nm bending motion (external rotation) during sequential cutting, as follows: LCL, PFL, and PT. IAR, instantaneous axis of rotation; +, values for the interior and posterior directions; −, values for the exterior and anterior directions. Values for the coordinate system are in millimeters

As shown in Fig. 6, the position of the IAR shifted medially and anteriorly after the ligaments and tendons had been reduced during external rotation under a 5 Nm bending motion. A slightly lateral shift in the trajectory of IAR was observed when the bending motion was increased to 10 Nm. LCL failure resulted in displacement of the IAR by 3.00 mm under 5 Nm bending motion and 3.02 mm under 10 Nm bending motion. Thus, under both 5 and 10 Nm external rotation, the simulated LCL failure caused the largest shift in the IAR.

## Discussion

The knee is one of the most complicated joints in the human body. Its three-dimensional movements include rotation and shift of the tibia and rolling of the femoral condyle. The surrounding ligaments and tendons stabilize the knee joint both in the static and dynamic state. The PLC consists of three layers of soft tissue. Failure of this complex can lead to external and varus instabilities of the knee. Previous anatomical and experimental studies demonstrated that the LCL, PFL, and PT play significant roles in maintaining the stability of the posterolateral knee. However, traffic accidents and sports injuries have gradually increased in frequency in daily life, as have injuries to the ligaments and tendons of the knee. PLC failure is primarily associated with high-energy trauma to the knee, which in most cases causes tibial plateau fracture or cruciate ligament injury. Tomás-Hernández et al. [24] reported a case series of large anteromedial tibial plateau fractures with PLC injury. Delee et al. [25] reported 12 cases of acute isolated posterolateral instability of the knee among 735 knee ligament injuries.

The clinical diagnosis of PLC injury of the knee can be difficult. Fanelli et al. [26] described three types of posterolateral instability, to help clinicians recognize PLC injury. Another study found that posterolateral pain of the knee can indicate PLC failure [27]. In a patient with a potential acute PLC injury, physical examination that includes the Lachman test should be performed [28]. Alternative diagnostic measures have also been proposed. Pritsch et al. [29] measured the TER in 14 fresh cadaver knees before and after transection of the medial collateral ligament. Based on their findings, the authors recommended the valgus stress test to aid diagnosis of posterolateral instability of the knee. For the evaluation of PLC injury, MRI has a clear advantage over X-ray and computed tomography, given its ability to image soft tissues. LaPrade et al. [7] used MRI for assessment of the PLC in 20 patients and concluded that it could accurately identify PLC injury. Ross et al. [6] found that MRI reliably depicted the extent of PLC injury preoperatively, based on observations of five complete PLC injuries and one partial tear.

Cadaver experiments have been widely used to investigate the biomechanics of the PLC. LaPrade et al. [30] assessed six fresh-frozen cadaver knees and determined that, under external rotation loads, the force on the LCL was higher than that on the PFL or PT. Due to its repeatability, biomechanical FE analysis has been applied in studies of the function and stability of the knee joint under various loads. Kang et al. [17] used the FE method to establish a musculoskeletal model and found that the forces acting on the anterior and posterior cruciate ligaments after PLC deficiency gradually increased.

In our FE model, several physical parameters (TD, TVA, TER, IAR) were measured during stepwise disruption of the PLC, to evaluate the stability of the knee joint under different failure conditions. For comparability with previous research, the order of PLC injury was LCL, PFL, and PT, and a 134 N anterior load was applied to the tibia. Peña et al. [17] reported that the tibial displacements were the 4.75 mm in the anterior direction, 0.56 mm in the medial direction, 1.10 mm in the distal direction, 0.76° in varus angulation, and 1.6° in external rotation under a combined anterior tibial load of 134 N and a compressive load of 1150 N. Gabriel et al. [15] also measured these five kinematics of the intact knee in response to a combined anterior tibial load of 134 N and a compressive load of 1150 N in their study. Similar results were obtained in our FE model. And the differences of distal translation and valgus rotation listed in Table 2 between Peña’s research and our study were probably due to the inclusion of PFL and PT. Therefore, our results were in good agreement with those studies. During sequential cutting of the PLC, the TD gradually increased, with the most significant shift occurring when the LCL was cut. This demonstrated the greater importance of the LCL than PFL and PT for maintaining anterior stability of the posterolateral knee.

Both the TVA and TER are crucial parameters in analyses of knee joint stability. In our study, pure bending motions of 5 and 10 Nm were applied to the tibia to achieve varus and external rotation during sequential cutting of the PLC. Chun et al. [18] evaluated the contributions of the LCL, PFL, and PT in twelve fresh-frozen cadaveric knees by sequentially cutting the PLC with four knee flexion of 0°, 30°, 60°, 90°, respectively. They illustrated that the PT and PFL contribute to the external rotatory stability equally, and the LCL is a significant structure to keep the varus rotatory stability. In the current study, the knee flexion was set 0°, and the sequential reduction of the PLC was set as follow: LCL, PFL, and PT. Both in 5 and 10 Nm varus rotation, the largest increment of TVA was observed after the LCL was cut. However, the increment of TVA was 0.46° under 5Nm bending motion, 2.09° under 10Nm bending motion, which were smaller than the increment after the reduction of PT. In external rotation, the largest increment of TER was also observed in the LCL reduction process. Contrary to varus rotation, the increment of TER after the reduction of PFL was observed larger than that in PT reduction process.

The location of the IAR has been used in analyses of segmental instability [31], but not in a FE model of PLC sequential injury. In varus rotation, the IAR shifted laterally and superiorly, toward the lateral femoral condyle, after PLC failure. On the contrary, the IAR during PLC sequential injury moved anteriorly and medially in external rotation. Thus, both in varus and in external rotation, the largest shift in IAR occurred when the LCL was cut. Displacement of the IAR was larger after PFL than PT disruption, except during 10 Nm external rotation.

The changes in TD, TVA, TER, and IAR determined in our study demonstrated the importance of the PLC in terms of both the stability of the posterolateral knee and consequences of dynamic injury. Within the PLC, the LCL played the largest role in maintaining stability. The current study may help the clinicians deepen their understanding of the PLC injury and assist them to offer better therapy to cure patients. However, the stability of the knee is complex, and there is still some controversy about the sequential injury order of the PLC. Furthermore, PLC injury is usually incidental to cruciate ligament failure or tibial plateau fracture. Thus, our results may have been influenced by the fact that our FE model did not include either a cut cruciate ligament or tibial plateau fracture.

## Conclusion

In our FE model, the TD became longer with sequential disruption of the PLC under a 134 N anterior load, with LCL disruption causing the greatest shift. Under both 5 and 10 Nm pure bending motion, the TVA and TER shifted in response to sequential damage of the PLC. The largest change occurred when the LCL was cut. However, while the IAR location changed when the PFL and PT were cut, a much larger shift occurred when the LCL was cut. The changes in TD, TVA, TER, and IAR during sequential damage of the PLC illustrate the importance of this complex structure for maintaining the stability of the knee, with the LCL being the most important structural component.

## Data Availability

The datasets used and/or analyzed during the current study are available from the corresponding author on reasonable request.

## Abbreviations

PLC: Posterolateral complex
LCL: Lateral collateral ligament
PFL: Popliteofibular ligament
PT: Popliteus tendon
FE: Finite element
TD: Tibial displacement
TER: Tibial external rotation
TVA: Tibial varus angulation
IAR: Instantaneous axis of rotation

## References

[1] Hughston J, Andrews J, Cross M, Moschi A (1976). Classification of knee ligament instabilities. Part II. The lateral compartment. JBJS..

[2] Terry GC, Laprade RF (1996). The posterolateral aspect of the knee. Anatomy and surgical approach. Am J Sports Med..

[3] Nannaparaju M, Mortada S, Wiiks A, Khan W, Alam M (2017). Posterolateral corner injuries: epidemiology, anatomy, biomechanics and diagnosis. Injury.

[4] Laprade RF, Wentorf F (2002). Diagnosis and treatment of posterolateral knee injuries. Clin Orthop Relat Res.

[5] Cohen AP, King D, Gibbon AJ (2001). Impingement fracture of the anteromedial tibial margin: a radiographic sign of combined posterolateral complex and posterior cruciate ligament disruption. Skelet Radiol.

[6] Ross G, Chapman AW, Newberg AR, Scheller AD (1997). Magnetic resonance imaging for the evaluation of acute posterolateral complex injuries of the knee. Am J Sports Med.

[7] Laprade RF, Gilbert TJ, Bollom TS, Wentorf F, Chaljub G (2000). The magnetic resonance imaging appearance of individual structures of the posterolateral knee. A prospective study of normal knees and knees with surgically verified grade III injuries. Am J Sports Med..

[8] Harish S, O’Donnell P, Connell D, Saifuddin A (2006). Imaging of the posterolateral corner of the knee. Clin Radiol..

[9] Zappia M, Oliva F, Chianca V, Di Pietto F, Maffulli N (2019). Sonographic evaluation of the anterolateral ligament of the knee: a cadaveric study. J Knee Surg.

[10] Castelli A, Zanon G, Jannelli E (2020). The role of the anterolateral ligament in knee's biomechanics: a case–control retrospective study. Eur J Orthop Surg Traumatol.

[11] Giovanni FT, Oliva F, Maffulli N (2017). Minimally invasive anatomic reconstruction of the anterolateral ligament with ipsilateral gracilis tendon. Muscles Ligaments Tendons J.

[12] Laprade RF, Ly TV, Wentorf FA, Engebretsen L (2003). The posterolateral attachments of the knee: a qualitative and quantitative morphologic analysis of the fibular collateral ligament, popliteus tendon, popliteofibular ligament, and lateral gastrocnemius tendon. Am J Sports Med.

[13] LaPrade RF, Resig S (1999). The effects of grade III posterolateral knee complex injuries on anterior cruciate ligament graft. Am J Sports Med..

[14] Peña E, Calvo B, Martínez MA, Doblaré M (2006). A three-dimensional finite element analysis of the combined behavior of ligaments and menisci in the healthy human knee joint. J Biomech.

[15] Gabriel MT, Wong EK, Woo LY, Yagi M, Debski RE (2004). Distribution of in situ forces in the anterior cruciate ligament in response to rotatory loads. J Orthop Res..

[16] Song Y, Debski RE, Musahl V, Thomas M, Woo SL (2004). A three-dimensional finite element model of the human anterior cruciate ligament: a computational analysis with experimental validation. J Biomech.

[17] Kang KT, Koh YG, Nam JH (2019). Biomechanical evaluation of the influence of posterolateral corner structures on cruciate ligaments forces during simulated gait and squatting. PLoS ONE..

[18] Chun Y-M, Kim S-J, Kim H-S (2008). Evaluation of the mechanical properties of posterolateral structures and supporting posterolateral instability of the knee. J Orthop Res.

[19] Orozco GA, Tanska P, Mononen ME, Halonen KS, Korhonen RK (2018). The effect of constitutive representations and structural constituents of ligaments on knee joint mechanics. Sci Rep.

[20] LaPrade RF (2005). Mechanical properties of the posterolateral structures of the knee. Am J Sports Med.

[21] Li YR, Gao YH, Yang C (2019). Finite-element analysis of the proximal tibial sclerotic bone and different alignment in total knee arthroplasty. BMC Musculoskelet Disord.

[22] Venäläinen M, Mononen ME, Jurvelin JS, Töyräs J, Virén T, Korhonen RK (2014). Importance of material properties and porosity of bone on mechanical response of articular cartilage in human knee joint–a two-dimensional finite element study. J Biomech Eng.

[23] Warnecke D, Meemer M, Roy LD (2019). Articular cartilage and meniscus reveal higher friction in swing phase than in stance phase under dynamic gait conditions. Sci Rep.

[24] Tomás-Hernández J, Monyart JM, Serra JT (2016). Large fracture of the anteromedial tibial plateau with isolated posterolateral knee corner injury: case series of an often missed unusual injury pattern. Injury.

[25] DeLee JC, Riley MB, Rockwood CA (1983). Acute posterolateral rotatory instability of the knee. J Bone Joint Surg Am.

[26] Fanelli GC, Larson RV (2002). Practical management of posterolateral instability of the knee. Arthrosc J Arthrosc Relat Surg.

[27] Dean RS, Laprade RF (2019). ACL and posterolateral corner injuries. Curr Rev Musculoskelet Med..

[28] Laprade RF, Terry GC (1997). Injuries to the posterolateral aspect of the knee: association of anatomic injury patterns with clinical instability. Am J Sports Med.

[29] Pritsch T, Blumberg N, Haim A, Dekel S, Arbel R (2006). The importance of the valgus stress test in the diagnosis of posterolateral instability of the knee. Injury.

[30] LaPrade RF (2004). Force measurements on the fibular collateral ligament, popliteofibular ligament, and popliteus tendon to applied loads. Am J Sports Med.

[31] Kim W, Espanha MM, Veloso AP, Araújo D, Kohles SS (2013). An informational algorithm as the basis for perception-action control of the instantaneous axes of the knee. J Nov Physiother.

